# The Vitamin E Analog Gamma-Tocotrienol (GT3) Suppresses Radiation-Induced Cytogenetic Damage

**DOI:** 10.1007/s11095-016-1950-0

**Published:** 2016-05-23

**Authors:** Rupak Pathak, Abdel Bachri, Sanchita P. Ghosh, Igor Koturbash, Marjan Boerma, Regina K. Binz, Jeffrey R. Sawyer, Martin Hauer-Jensen

**Affiliations:** 1 Division of Radiation Health, Department of Pharmaceutical Sciences, College of Pharmacy, University of Arkansas for Medical Sciences, Biomed I, Suite 238, 4301 West Markham, Slot 522-3, Little Rock, Arkansas 72205 USA; 2 Department of Engineering and Engineering Physics, Southern Arkansas University, Magnolia, Arkansas USA; 3 Armed Forces Radiobiology Research Institute, USUHS, Bethesda, Maryland USA; 4 Department of Environmental and Occupational Health, University of Arkansas for Medical Sciences, Little Rock, Arkansas USA; 5 Department of Pathology, University of Arkansas for Medical Sciences, Little Rock, Arkansas USA; 6 Central Arkansas Veterans Healthcare System, Little Rock, Arkansas USA

**Keywords:** chromosomal aberrations, DNA repair, endothelial cells, gamma-tocotrienol, ionizing radiation

## Abstract

**Purpose:**

Ionizing radiation (IR) generates reactive oxygen species (ROS), which cause DNA double-strand breaks (DSBs) that are responsible for cytogenetic alterations. Because antioxidants are potent ROS scavengers, we determined whether the vitamin E isoform γ-tocotrienol (GT3), a radio-protective multifunctional dietary antioxidant, can suppress IR-induced cytogenetic damage.

**Methods:**

We measured DSB formation in irradiated primary human umbilical vein endothelial cells (HUVECs) by quantifying the formation of γ-H2AX foci. Chromosomal aberrations (CAs) were analyzed in irradiated HUVECs and in the bone marrow cells of irradiated mice by conventional and fluorescence-based chromosome painting techniques. Gene expression was measured in HUVECs with quantitative reverse transcriptase polymerase chain reaction (qRT-PCR).

**Results:**

GT3 pretreatment reduced DSB formation in HUVECS, and also decreased CAs in HUVECs and mouse bone marrow cells after irradiation. Moreover, GT3 increased expression of the DNA-repair gene *RAD50* and attenuated radiation-induced *RAD50* suppression.

**Conclusions:**

GT3 attenuates radiation-induced cytogenetic damage, possibly by affecting *RAD50* expression. GT3 should be explored as a therapeutic to reduce the risk of developing genetic diseases after radiation exposure.

## Introduction

Ionizing radiation (IR) compromises the redox state of cells and generates reactive oxygen species (ROS) in excess. ROS are highly reactive and damage DNA, as well as various cellular components. Mammalian cells exposed to 1 Gy of IR accumulate approximately 1000 single-strand breaks, 40 double-strand breaks (DSBs), and 3000 damaged DNA bases (1). DSBs are serious lesions that can cause numerous structural chromosomal aberrations (CAs) such as sister chromatid unions, chromatid- or chromosome-type breaks, and dicentric or ring chromosomes (2). Such CAs are particularly deleterious and are associated with the progression and development of several diseases, including cancer (3). To date, CAs have been found in all major tumor types and are also associated with mental illness, congenital heart disease, and respiratory problems (4–6). This suggests that CAs are not only a hallmark of cancer but also a cytogenetic signature of various pathophysiological conditions. While CAs can arise spontaneously, radiation is a major factor that induces CAs.

DNA-repair proteins such as RAD50 mitigate CAs by repairing DSBs. Curiously, radiation suppresses *RAD50* gene expression in mouse white blood cells for up to 48 h after irradiation (7). RAD50 is part of the MRN complex which consists of meiotic recombination 11 (MRE11), RAD50, and Nijmegen breakage syndrome 1 (NBS1) proteins. The primary function of the MRN complex is to recognize DSBs, bind the damaged DNA, and initiate repair by regulating the homologous recombination pathway and the alternate-non-homologous end- joining cascade with support from other DNA-repair proteins (8).

The type and frequency of radiation-induced CAs vary with radiation dose, dose rate, and the quality of irradiation. CAs may be evident in metaphase chromosomes soon after irradiation, or they may appear several generations later. This delay is a sign of genomic instability and is a hallmark of cancer (9). Similarly, IR is a major risk factor for endothelial dysfunction (10), and genetically unstable endothelial cells can directly stimulate cancer metastases (11,12). Thus, both cancer and endothelial dysfunction may benefit from therapies that reduce radiation-induced oxidative stress.

Antioxidants suppress DSBs and CAs in irradiated human lymphocytes (13,14). The vitamin E isoform γ-tocotrienol (GT3) is a natural dietary antioxidant and a potent radio-protector, with the largest dose reduction factor among all the natural products identified to date (15,16). GT3 protects the endothelials cells that line the blood and lymphatic vessels of higher eukaryotes (17), regulates a number of signaling pathways that mitigate radiation damage (18), and also protects mice from a lethal dose of radiation (16). Although we do not fully understand how GT3 protects against radiation damage, studies by various groups, including our own, suggest that GT3 may regulate apoptotic signals, cytokine production, cholesterol biosynthesis, or progenitor cell mobilization (17,19–21). Moreover, gene-expression profiles suggest that GT3 affects a number of genes known to be important for the DNA damage response (18).

Here, we demonstrate that GT3 pretreatment decreases the frequency of radiation-induced DSBs and CAs both *in vitro* and *in vivo.* We also show that GT3 stabilizes *RAD50* expression after irradiation. These findings suggest that GT3 is a potent radio-protector that can protect against radiation-induced cytogenetic damage.

## Materials and Methods

### Materials

GT3 was obtained from Yasoo Health Inc. (Johnson City, TN). The EGM-2 Bullet Kit and HEPES-buffered saline solution used for cell culture were obtained from Lonza (Walkersville, MD). All other reagents and media were obtained from Sigma (St. Louis, MO).

### Cell Culture and Animals

Primary human umbilical vein endothelial cells (HUVECs) (American Type Culture Collection, Manassas, VA) were cultured in tissue culture vessels with EGM-2 Bullet Kit in 5% CO_2_ incubator at 37°C. The cells were passaged every 2 to 3 days with standard aseptic techniques, and experiments were performed using cells from passages 3 to 6.

All animal studies were carried out in strict accordance with the Guide for the Care and Use of Laboratory Animals of the National Institutes of Health. The animal protocol was approved by the Institutional Animal Care and Use Committee of the University of Arkansas for Medical Sciences. Female C57BL/6J mice (The Jackson Laboratory, Bar Harbor, ME, stock no. 000664) were held in quarantine for 1 week after arrival. All animals were housed in conventional cages in a standard air conditioned animal facility at 20 ± 2°C, with 10–15 hourly cycles of fresh air, and a 12–12-h day-night light cycle. Animals had free access to water and chow (Envigo Teklad diet 7012, Madison, WI). All animals were humanely euthanized with CO_2_ in an appropriate chamber.

### Irradiation and Drug Treatment

Mice were irradiated with a single, total-body radiation dose (3 Gy) in a Shepherd Mark I model 25 ^137^Cs irradiator (J. L. Shepherd & Associates, San Fernando, CA). The average dose rate was 0.98 Gy/min and was corrected for decay. The animals were placed in a well-ventilated rotating chamber composed of T-6061 aluminum with a gold-anodized coating. The chamber was divided into eight equal “pie slice” compartments with a well-ventilated Plexiglas lid (J.L. Shepherd & Associates). Animals were injected subcutaneously into the loose skin over the neck with a single dose of GT3 or vehicle (5% Tween-80 in saline) 24 h before irradiation. All radiation experiments were performed in the morning to minimize diurnal effects. HUVECs were irradiated in T25 tissue-culture flasks (Corning, NY) or chambered slides (Nunc Lab-Tek, St. Louis, MO). HUVECs were treated with 0–5 μM of GT3 or vehicle (DMSO) prior to irradiation. Radiation doses used for the various studies were carefully selected based on experimental endpoints and knowledge regarding cellular radio-sensitivity.

### γ-H2AX Detection

HUVECs were grown in 4-well chambered slides overnight and treated with GT3 or vehicle (DMSO) for 1 h or 24 h before irradiation. Cells were washed with PBS and fixed with 4% paraformaldehyde 1 h after irradiation. The fixed cells were washed, permeabilized with 0.3% Triton-X for 15 min at room temperature, quenched with 1% glycine, and blocked with 4% BSA in 1X TBST overnight at 4°C. The cells were incubated with primary antibody (1:600 dilution; monoclonal Anti-phospho-Histone H2AX (Ser139), clone JBW301; Millipore, Temecula, CA) for 3 h at room temperature. After washing 3 times with PBS, secondary antibody (Alexa Fluor 594 F (ab') 2 fragment of goat anti-mouse IgG; Invitrogen, Grand Island, NY) was added, and the cells were incubated for 1 h at room temperature in the dark. Finally, the cells were washed with PBS and stained with Hoechst solution to visualize nuclei. γ-H2AX foci were detected with a Zeiss AxioPlan microscope. γ-H2AX foci were not counted in apoptotic cells. The apoptotic cells, particularly cells in late apoptotic stages, were identified by their highly compromised nuclear membrane.

### Preparation of Metaphase Spreads

For *in vitro* experiments, HUVECs were treated with 5 μl/ml colcemid (Invitrogen, Grand Island, NY) 24 h before cell harvest. HUVECs were collected by trypsinization and washed 2 times with PBS. For *in vivo* experiments, mice were given 100 μl of 0.05% colchicine intraperitoneally 2 h before bone marrow harvest. Bone marrow cells were collected from the tibias and femurs of both hind legs by flushing with ice cold PBS containing 2% FBS. The cell suspension was layered onto equal volume (3 ml) of Histopaque-1083 (Sigma-Aldrich, St. Louis, MO) in a 15-ml tube. The tubes were centrifuged at 400 *g* for 30 min at room temperature. The buffy coat, containing bone marrow mononuclear cells, was collected and washed with PBS. HUVECs and mouse bone marrow mononuclear cells were then treated with hypotonic solution (0.56% KCl solution) for 15 min and fixed in methanol-acetic acid (3:1). Fixed cells were applied to precleaned, wet glass slides and air-dried overnight at room temperature.

### Chromosome Staining Techniques

Two techniques were used to examine CAs in metaphase spreads. Giemsa staining: Metaphase spreads were prepared as described above and air dried at room temperature overnight. Slides were immersed in Giemsa staining solution (Fisher Scientific) for 20 min, rinsed in distilled water to remove excess stain, and air-dried for 24 h before scoring at 100x magnification under bright field on a Zeiss microscope. Multiple fluorescence *in situ* hybridization (mFISH) painting: Whole-chromosome DNA probes (Applied Spectral Imaging, Carlsbad, CA) were used to label human and mouse chromosomes 1, 2, and 3, according to the manufacturer’s instructions. Metaphase spreads were prepared 24 h before mFISH painting. The slides were treated with 2X SSC buffer at room temperature for 2 min, then dehydrated in a series of ethanol solutions (70%, 80%, and 100%) for 2 min each. Chromosomes were denatured by placing the slides in pre-warmed (72°C) denaturation solution (70% formamide in 2X SSC) for 60–80 s, then immediately passed through a series of cold ethanol solutions (70%, 80%, and 100%) and air-dried. mFISH probe (10 μl) was denatured at 80°C for 7 min and applied to the slides. The slides were covered with glass cover slips, sealed with rubber cement, and incubated overnight at 37°C in a humidified CO_2_ incubator. The coverslips were removed, and the slides were washed with 0.4X SSC at 74°C for 5 min and placed in washing solution-II (4X SSC with 0.1% Tween-20) for 2 min. Finally, the slides were counter-stained with 10 μl of DAPI containing anti-fade (Vector Laboratories, Burlingame, CA) and sealed with a coverslip.

### qRT-PCR

HUVECs were washed twice with cold calcium- and magnesium-free PBS. Total RNA was extracted with the RNeasy Plus Mini Kit (Qiagen, Valencia, CA), and each sample was treated with TURBO DNase (Ambion, Grand Island, NY). RNA was quantified with an Agilent 2100 Bioanalyzer (Agilent Technologies, Santa Clara, CA). Samples with an RNA integrity number (RIN) of 10 were used to produce cDNA. Although in a typical *in vitro* experiment RIN value varies between 7.5 and 10, in the current study we used samples with RIN value 10 to ensure reproducibility and accuracy of the gene expression study. cDNA was generated from 2 μg of RNA using the high-capacity cDNA Reverse Transcription Kit (Applied Biosystems, Carlsbad, CA). qRT-PCR was performed with the ABI Prism 7000 Sequence Detection System (Applied Biosystems). Standard real-time PCR (50°C for 2 min; 95°C for 10 min; 50 cycles of 95°C for 15 s, 60°C for 60 s) was performed with TaqMan 2X Universal PCR Master Mix (Applied Biosystems). Relative mRNA expression was calculated with the comparative *C*_T_ method (2^-∆∆*C*T^) and normalized to 18S rRNA for each sample.

### Statistical Analysis

Statistical analyses were performed with GraphPad software (La Jolla, CA). For experiments with only one parameter under consideration, *p* values were determined by an unpaired Student’s *t* test. The standard error for CAs was calculated by √*a/A*, where *a* was the number of aberration under consideration and *A* was the total number of cells analyzed (22). *Z* tests were used to test differences in the frequency of CAs before and after exposure to IR, as described elsewhere (23). Results were considered statistically significant when *p* < 0.05.

## Results

### GT3 Suppresses Radiation-Induced DSB Formation in Primary Human Endothelial Cells

**Fig. 1:**
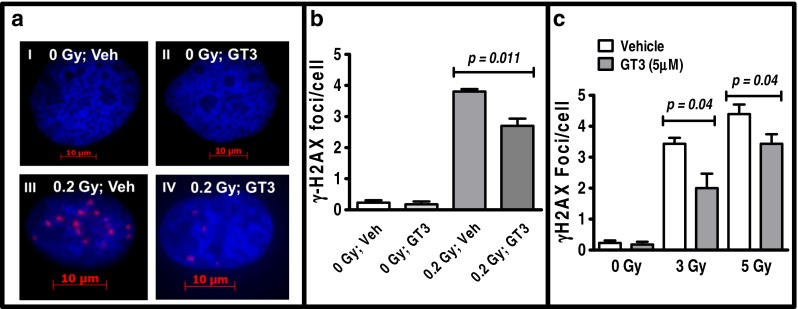
GT3 decreases the number of radiation-induced γ-H2AX foci (*red*) in HUVEC nuclei (DAPI stain, *blue*). (**a**) HUVECs were treated with (*I*) vehicle (Veh, DMSO) for 24 h or (*II*) 5 μM GT3 for 24 h without irradiation, or (*III*) pretreated with vehicle or (IV) 5 μM GT3 24 h before 0.2 Gy irradiation. Images were taken 1 h after irradiation. (**b**) Data from (**a**) are expressed as γ-H2AX foci per cell for each treatment. (**c**) To determine the efficacy of GT3 in suppressing persistent DSBs, HUVECs were treated with GT3 or vehicle for 1 h, exposed to 0, 3 and 5 Gy of radiation and further incubated for 29 h before γH2AX immunofluorescence staining. At least 200 nuclei were counted per treatment group. The data are presented as the mean ± SEM of 3 to 6 independent assays and *p* values were determined by an unpaired Student’s *t* test.

Because GT3 has strong antioxidant properties and protects endothelial cells from radiation damage (15,17), we proposed that GT3 pretreatment would suppress radiation-induced DSBs in HUVECs. To determine whether GT3 was able to suppress DSBs in HUVECs, we assayed the formation of γ-H2AX foci as an indicator of DSBs after irradiation. Previous gene expression profiling study from our group show that GT3 treatment for 24 h substantially alters genetic pathways known to be of critically important in the regulation of cellular responses of radiation exposure, including the responses to DNA damage stimuli in HUVECs (18). However, a recent study demonstrate GT3 treatment for 1 h considerably suppresses AKT phosphorylation, another critical marker of DSBs formation, in HUVECs (24). Therefore, we decided to treat HUVECs with GT3 either for 1 h or 24 h to assess its ability to modify radiation-induced γH2AX foci formation. HUVECs were treated with either 5 μM GT3 or vehicle (DMSO) 24 h before irradiation with 0.2 Gy of γ-rays. γ-H2AX foci were quantified by microscopy 1 h after irradiation (a low dose of radiation was used to minimize counting error). γ-H2AX foci were almost undetectable in non-irradiated cells treated with vehicle or GT3 (Fig. 1a I, II, b). Irradiation induced γ-H2AX foci in both the vehicle- (3.8 ± 0.07) and GT3-treated groups (2.7 ± 0.23) but the GT3-treated cells contained significantly (*p* = 0.01) fewer γ-H2AX foci (Fig. 1a III, VI, b). Further, to determine the ability of GT3 in suppressing residual DSBs that are persisted several hours after irradiation, we treated HUVECs with 5 μM of GT3 for 1 h, then exposed to two different radiation doses 3 Gy and 5 Gy, and further incubated for 29 h before immunofluorescence staining for γH2AX foci. We observed GT3 also effectively suppressed frequency of persistent DSB formation in primary human endothelial cells after 3 Gy (3.4 ± 0.19 in vehicle treated group vs 2.0 ± 0.46 in GT3 treated group) and 5 Gy (4.4 ± 0.31 in vehicle treated group vs 3.2 ± 0.23 in GT3 treated group) irradiation (Fig. 1c).

### GT3 Decreases Chromosomal Aberrations in Irradiated Primary Human Endothelial Cells

Next, we asked whether GT3 pretreatment could decrease CAs in irradiated endothelial cells. HUVECs were irradiated with 5 Gy, and CAs were visualized with Giemsa staining and mFISH analysis with probes specific for human chromosomes 1, 2, and 3. CAs were scored as chromatid-type breaks, sister chromatid unions, chromatid-type exchanges, dicentric and ring chromosomes, double minutes, and chromosome-type breaks. The percentage of radiation-induced CAs was lower in HUVECs pretreated with GT3 (115.6 ± 13.4) than in the vehicle-treated group (176.3 ± 15.2), as detected by Giemsa staining (Table I). Likewise, the number of radiation-induced CAs in chromosomes 1, 2, and, 3 was lower in HUVECs pretreated with GT3 (90.5 ± 11.1) than in the vehicle-treated group (128.4 ± 10.5) (Table II). Thus, GT3 effectively decreased DSBs and CAs in HUVECs.

**Table I:** Percentages of CAs in HUVECs. Aberration Percentage ± SE (Total Number of Aberration Observed) in Metaphase Spreads of HUVECs After Various Treatment As Detected By Conventional Giemsa Solid Staining. Standard Errors on the Aberration Percentage were Calculated By √a/A, As Described in Material and Method Section. The Abbreviations Used for CAs Are As Follows CTB, Chromatid-Type Break; SCU, Sister Chromatid Union; CTE, Chromatid-Type Exchange; DIC/R, Dicentric/Ring; DIMN, Double Minutes; CSB, Chromosome-Type Break

Treatment	Normal metaphase	Aberrant metaphase	CTB	SCU	CTE	DIC/R	DIMN	CSB	Total aberration
Vehicle; 0 Gy	99.4 ± 8.0 (155)	0.6 ± 0.6 (1)	0.6 ± 0.6 (1)	0.0 ± 0.0 (0)	0.0 ± 0.0 (0)	0.0 ± 0.0 (0)	0.0 ± 0.0 (0)	0.0 ± 0.0 (0)	0.6 ± 0.6 (1)
GT3; 0 Gy	100.0 + 10.0 (100)	0.0 + 0.0 (0)	0.0 + 0.0 (0)	0.0 + 0.0 (0)	0.0 + 0.0 (0)	0.0 + 0.0 (0)	0.0 + 0.0 (0)	0.0 + 0.0 (0)	0.0 + 0.0 (0)
Vehicle; 5 Gy	31.6 ± 6.4 (24)	68.4 ± 9.5 (52)	114.5 ± 12.3 (87)	9.2 ± 3.5 (7)	3.9 ± 2.3 (3)	21.1 ± 5.3 (16)	2.6 ± 1.9 (2)	25.0 ± 5.7 (19)	176.3 ± 15.2 (134)
GT3; 5 Gy	48.4 ± 8.7 (31)	51.6 ± 9.0 (33)	85.9 ± 11.6 (55)	6.3 ± 3.1 (4)	0.0 ± 0.0 (0)	12.5 ± 4.4 (8)	0.0 ± 0.0 (0)	10.9 ± 4.1 (7)	115.6 ± 13.4 (74)

**Table II:** Percentages of CAs in HUVEC Chromosomes 1, 2, and 3. Aberration Percentage ± SE (Total Number of Aberration Observed) in the Metaphase Spreads of HUVECs After Various Treatment As Detected by mFISH Using Whole Chromosome DNA Probe for Human Chromosome 1, 2, and 3. Standard Errors on the Aberration Percentage Were Calculated By √a/A, As Described in Material And Method Section

Treatment	Normal metaphase	Aberrant metaphase	Chr-1	Chr-2	Chr-3	Total aberrations
Vehicle; 0Gy	97.1 ± 7.6 (165)	2.9 ± 1.3 (5)	0.6 ± 0.6 (1)	1.2 ± 0.8 (2)	1.8 ± 1.0 (3)	3.5 ± 1.4 (6)
GT3; 0Gy	96.5 ± 7.5 (167)	3.5 ± 1.4 (6)	1.2 ± 0.8 (2)	0.6 ± 0.6 (1)	1.7 ± 1.0 (3)	3.5 ± 1.4 (6)
Vehicle; 5Gy	38.8 ± 5.8 (45)	61.2 ± 7.3 (71)	49.1 ± 6.5 (57)	35.3 ± 5.5 (41)	44.0 ± 6.2 (51)	128.4 ± 10.5 (149)
GT3; 5Gy	52.7 ± 8.4 (39)	47.3 ± 8.0 (35)	39.2 ± 7.3 (29)	21.6 ± 5.4 (16)	29.7 ± 6.3 (22)	90.5 ± 11.1 (67)

### GT3 Decreases Chromosomal Aberrations in Irradiated Mice

**Fig. 2:**
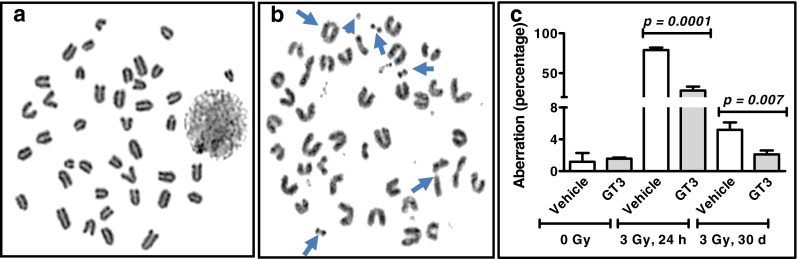
GT3 decreases radiation-induced CAs in mouse bone marrow cells. CAs were assayed by Giemsa solid staining in mouse bone marrow metaphase spreads from (**a**) non-irradiated (**b**) or irradiated mice (CAs indicated by *arrows*). (**c**) Mice (*n* = 3) were pretreated with vehicle or GT3 (200 mg/kg) 24 h before 3 Gy of total body irradiation. CAs were scored in 200–600 metaphase spreads 24 h and 30 days after irradiation. Standard deviation represents interindividual (*n* = 3) variability. An unpaired *t* test was performed to determine *p* values.

**Fig. 3:**
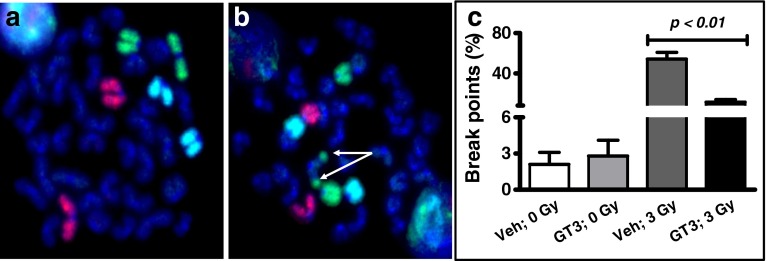
GT3 decreases radiation-induced CAs in mouse chromosomes 1 (*red*), 2 (*green*), and 3 (*blue*). CAs were assayed by mFISH in metaphase spreads from (**a**) non-irradiated or (**b**) irradiated mice (CAs indicated by *arrows*). (**c**) Mice (*n* = 3) were pretreated with vehicle or GT3 (200 mg/kg) 24 h before 3 Gy of total-body irradiation and pooled bone marrow samples were assayed by mFISH. CAs were scored in 100–300 metaphase spreads and the standard error was calculated by √*a/A* as described in materials and method section.

Considering that GT3 effectively decreased CAs *in vitro*, we predicted that GT3 would decrease CAs in mice after irradiation. Mice were administered GT3 (100 μl at dose of 200 mg/kg) subcutaneously 24 h before 3 Gy of total-body irradiation, and bone marrow mononuclear cells were collected 24 h or 30 days after irradiation. We used a relatively lower dose (3 Gy) for the *in vivo* study than for the *in vitro* chromosomal aberration assay (5 Gy) because lymphocytes are more radiosensitive than HUVECs. Giemsa staining detected CAs in mouse bone marrow cells 24 h after irradiation (Fig. 2b, arrows), compared to the non-irradiated control (Fig. 2a). However, GT3 suppressed CAs in the irradiated bone marrow cells both 24 h (*p* = 0.0001) and 30 days (*p* = 0.007) after irradiation (Fig. 2c). GT3 treatment also significantly (*p* < 0.01) decreased radiation-induced CAs involving mouse chromosomes 1, 2, and 3 24 h after irradiation (Fig. 3). Thus, GT3 effectively decreased CAs *in vivo* for up to 30 days after total-body irradiation.

### GT3 Increases *RAD50* Expression

**Fig. 4:**
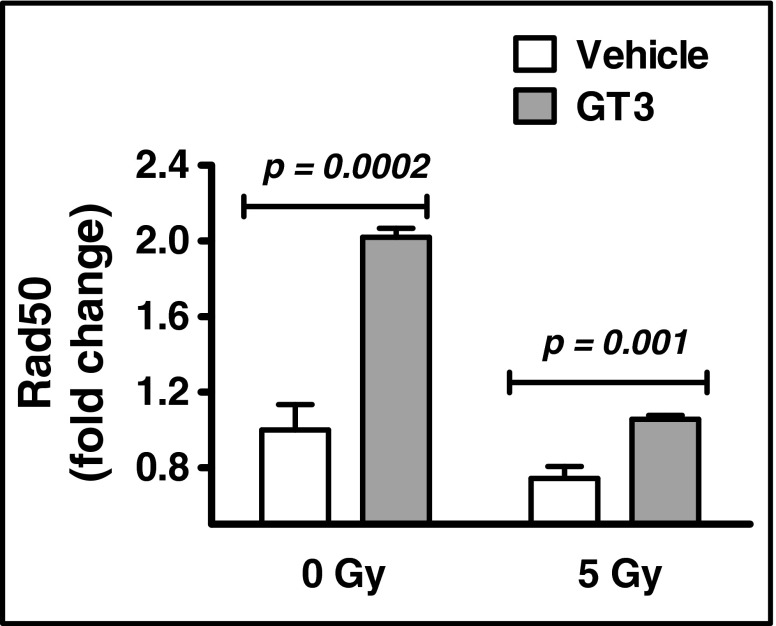
GT3 modulates *RAD50* expression. HUVECs were treated with vehicle or GT3 (5 μM) for 1 h before 0 Gy and 5 Gy irradiation, cells were further incubated for 23 h before harvest. *RAD50* mRNA was measured relative to 18S rRNA with Taqman assay. The data are presented as the mean ± SD of three independent assays.

Because GT3 decreased the incidence of DSBs *in vitro*, we reasoned that GT3 may affect expression of the DSB-repair protein RAD50. *RAD50* expression in GT3-treated HUVECs was roughly two-fold higher than in vehicle-treated cells (Fig. 4). Irradiation is known to decrease *RAD50* expression, and this was, indeed, the case for both GT3- and vehicle-treated cells (Fig. 4); however, GT3 maintained *RAD50* expression at a level similar to that observed in non-irradiated, vehicle-treated controls (Fig. 4). These findings suggest that GT3 restricts radiation-induced cytogenetic damage, possibly by modulating *RAD50* expression.

## Discussion

Radiation generates ROS that often cause DSBs. Studies from various groups, including our own, suggest that GT3 detoxifies ROS, thereby expecting it to reduce DSB formation. Here, we demonstrate that GT3 suppresses DSB formation in irradiated HUVECs. Specifically, GT3 reduced the number of γ-H2AX foci, a strong indicator of DSBs, by roughly 29% in HUVECs. We suggest several mechanisms to explain these findings. GT3 may regulate mitochondrial activity, transcription factors, or endothelial cell surface receptors. For example, GT3 alleviates ROS by preventing mitochondrial dysfunction in primary rabbit renal tubular cells exposed to *tert*-Butyl hydroperoxide (25). With respect to transcription factors, GT3 decreases ROS in human oral keratinocytes exposed to chemotherapy by stabilizing Nrf2 activation. Nrf2 is a transcription factor that regulates cellular antioxidant pathways (26). Further, we recently demonstrated that GT3 increases the expression and activity of the endothelial surface receptor thrombomodulin (TM) (27). The lectin-like domain of TM activates Nrf2-dependent antioxidant pathways, substantially decreasing serum and renal ROS levels in a mouse model of type-2 diabetic nephropathy (28). Moreover, TM forms a complex with the coagulation factor thrombin to generate activated protein C (APC) in the blood (29). Among its many properties, APC prevents glucose-induced oxidative stress in endothelial cells (30). Thus, our data indicate that GT3 protects HUVECs from ROS-mediated DSBs, and it may do so in a number of ways.

Improperly repaired DSBs induce CAs that are visible in metaphase chromosomes. We found that GT3 decreases CAs in both irradiated HUVECs and primary mouse bone marrow cells. It is not entirely clear how GT3 protects cells from induction of CAs after radiation exposure, but we offer one possible explanation. GT3 induces the production of granulocyte colony stimulating factor (G-CSF) in mice (31) and evidence suggests that G-CSF reduces chromosomal translocations in patients with cancer (32). Specifically, G-CSF prevented translocations between the long arms of chromosomes 8 and 21 in patients with acute myeloid leukemia (32). The role of G-CSF may not be so straightforward, however. G-CSF was shown to cause aneuploidy in healthy individuals (33), although a separate study found no such evidence (34).

Regardless of the specific mechanisms involved, data suggest that various vitamin E isoforms, including GT3, protect against cytogenetic damage in humans. For example, α-tocopherol decreases the frequency of chromatid-type breaks and sister chromatid exchanges in lymphocytes from people with Fanconi anemia (35,36). Similarly, α-tocopherol decreases the accumulation of CAs during G_2_ phase in Down’s syndrome patients (37). Finally, a commercially available, tocotrienol-rich vitamin E derivative decreases sister chromatid exchanges in elderly human subjects (38). Thus, multiple vitamin E isoforms could be used to prevent radiation-induced cytogenetic damage in endothelial cell.

Finally, intracellular DNA-repair enzymes are critically important for repairing cytogenetic damage. Radiation damage activates the cellular DNA-repair machinery that identifies DSBs and recruits DNA-repair proteins. Previously, we found that GT3 modulates as many as 27 gene clusters in HUVECs involved in DNA damage (18). Here, we show that GT3 increases *RAD50* expression and attenuates the radiation-induced suppression of *RAD50*. These findings corroborate a previous study by Li *et al.* that demonstrated that *Rad50* expression was suppressed for up to 48 h in mouse white blood cells after 8-Gy irradiation (7). Experimental evidence suggests that not only is RAD50 required for efficient DNA repair after irradiation (39) but also that its deficiency makes early embryonic cells hypersensitive to IR (40).

## Conclusion

The present studies demonstrate that GT3 pretreatment decreases radiation-induced DSB and CA formation in HUVECs. GT3 also reduces acute and delayed CAs in irradiated mice and increases *RAD50* expression in HUVECs. We conclude that GT3 suppresses radiation-induced cytogenetic damage and possibly GT3-mediated alteration in Rad50 expression plays some role in limiting cytogenetic damage after radiation; however, further investigation is required to determine whether GT3 regulates additional DNA-repair proteins. GT3 may, therefore, be a useful therapeutic to protect against radiation-induced genetic diseases.

## Abbreviations

APC: Activated protein C
CAs: Chromosomal aberrations
DMSO: Dimethyl sulfoxide
DSBs: DNA double strand breaks
EPCR: Endothelial protein C receptor
G-CSF: Granulocyte colony stimulating factor
GT3: γ-tocotrienol
Gy: Gray unit
HUVECs: Human umbilical vein endothelial cells
IR: Ionizing radiation
mFISH: Multiple fluorescence *in situ* hybridization
qRT-PCR: Quantitative reverse transcriptase polymerase chain reaction
Rad50: RAD50 homolog, double strand break repair protein
ROS: Reactive oxygen species
TM: Thrombomodulin
